# Impact of heavy metals on antibiotic resistance of *Escherichia coli* from slum wastewater in Kawempe division, Kampala district, Uganda: a case study

**DOI:** 10.1186/s12866-025-04024-1

**Published:** 2025-05-21

**Authors:** Isaac Byarugaba, Alice Nabatanzi, Emmanuel Muhumuza, Joseph Kyambadde

**Affiliations:** 1 Department of Biochemistry and Systems Biology, School of Biosciences, College of Natural Sciences, Makerere University, P. O. Box 7062, Kampala, 00256 Uganda; 2 Department of Plant Sciences, Microbiology and Biotechnology, School of Biosciences, College of Natural Sciences, Makerere University, P. O. Box 7062, Kampala, 00256 Uganda

**Keywords:** Antibiotic resistance, *Escherichia coli*, Heavy metals, Slums, Wastewater

## Abstract

**Background:**

Slum dwellers face significant infrastructure and public health challenges like poor housing and drainage, inadequate sanitation, and limited access to clean water, leading to increased disease transmission and resistance to antibiotic treatments. This study evaluated the impact of heavy metals on antibiotic resistance patterns of *Escherichia coli* in wastewater from slums of Bwaise II, Bwaise III, Kazo, and Makerere III in Kawempe division, Kampala.

**Methods:**

Levels of heavy metals (lead, mercury, cadmium, chromium, and arsenic) in wastewater were determined using inductively coupled plasma mass spectroscopy. *Escherichia coli* were isolated from wastewater using MacConkey agar and their susceptibility to 50 µl of stock antibiotics (tetracycline, amoxicillin, ceftriaxone at 30 µg/ml, and ciprofloxacin at 5 µg/ml) determined. The potential of heavy metals to induce antibiotic resistance in *Escherichia coli* was determined by culturing susceptible isolates in 200 µl of Luria-Bertina broth containing stock antibiotics (10 µl), or stock antibiotics (10 µl) and stock heavy metals (10 µl). Stock heavy metals were prepared from the average concentration of heavy metals detected in wastewater.

**Results:**

Detectable levels of heavy metals were reported in wastewater from Bwaise II, Kazo and Makerere III only. Lead, cadmium and arsenic, mercury and chromium, were highest in Bwaise II, Kazo, and Makerere III, respectively. The occurrence of *Escherichia coli* resistant to at least an antibiotic was 72.8% (169 of 232) and resistance to tetracycline, ceftriaxone, amoxicillin, and ciprofloxacin were 34.1%, 28.9%, 35.3%, and 34.5%, respectively. Study findings further revealed a positive correlation (R^2^ = 0.371–0.985) between the presence of heavy metals in wastewater and antibiotic resistance patterns of *Escherichia coli*. Also, heavy metals; lead (77.41 µg/ml), mercury (1.44 µg/ml), and cadmium (10.21 µg/ml) significantly (*p* < 0.05) induced antibiotic resistance in susceptible *Escherichia coli*.

**Conclusion:**

Wastewater in Kawempe slums is polluted with heavy metals and high prevalence of antibiotic-resistant *Escherichia coli.* Inadequate infrastructure in slums facilitate discharge of wastewater polluted with heavy metals, which in turn play a role in increasing antibiotic resistance. There is need for proper wastewater management to contain the prevalence of antibiotic resistance.

## Background

Population growth and rural-urban migration in pursuit of employment opportunities, better living standards and services have resulted in urban sprawl [1, 2]. This has contributed to the increase in slum residents [3]. About 863 million people of the global population and over half of the urban population in lower-income countries are residents of slums [4]. Approximately 53.6% of the people in Uganda’s capital city Kampala are living in slum communities [5]. Slum dwellers face significant infrastructure and public health challenges such as inadequate sanitation, poor housing and drainage, limited access to clean water, which in turn lead to increased disease transmission and resistance to antibiotic treatments [6]. Consequently, residents are at risk of suffering from life-threatening waterborne and soil-hosted diseases such as diarrhea, typhoid, and cholera [7]. In developing countries, about 88% of diarrhea cases are attributed to inadequate hygiene and the use of contaminated water [8]. Moreover, *Escherichia coli* (*E. coli*) a member of fecal coliforms, yet an important pathogen for diarrhea is most prevalent in such environmental conditions [9].

Water contamination is caused by industrial, domestic, agricultural and radioactive wastes [10, 11]. These wastes enter the water system through leaking sewers, urban and agricultural runoffs, industrial and household wastewater, often contaminated with various bacteria and unquantifiable quantities of antibiotic resistance driving agents such as residual antibiotics, disinfectants, heavy metals and other toxic chemicals [12, 13]. These in turn favor microbial growth and establish antibiotic selection pressure which drives the proliferation of antibiotic resistance in pathogenic microorganisms, such as *E. coli*, leading to a rise in the prevalence of antibiotic-resistant bacteria in water systems [14–18]. Antibiotic resistance is associated with reduced treatment efficacy and options, high medical costs, recurring health complications, and prolonged hospital stays, hence increased mortality rate [19, 20]. As a matter of concern, the World Health organization (WHO) has recognized antibiotic resistance as one of the top environmental health challenges facing humanity to date with the global mortality rate estimated at 700,000 deaths per year [21–23]. Antibiotic resistance occurs when bacteria develop mechanisms to evade antibiotic effects allowing them to persist and spread despite treatment [24]. These mechanisms include; chromosomal mutations in genes coding for the antibiotic targets, over-expression of efflux pumps, physical blocking of the antibiotic targets, and enzymatic modification of the antibiotics [25–29].

Studies by [30–33], reported a correlation between heavy metals and antibiotic resistance in point sources of contamination, i.e., wastewater treatment plants, animal farms, solid waste dump sites, and health facilities, respectively. Studies on determining the levels of heavy metal in non-point sources of contamination such as in slum communities and their effects on microbial communities are still limited. In addition, majority of studies aimed at understanding the causes of antimicrobial resistance were limited to the misuse of antibiotics which is associated with increased antibiotic residue pollution. However, it is possible that chemical contaminants discharged in the environment could have a significant effect on the abundance of resistance genes and proliferation of antibiotic selection pressure [34].

In Uganda, no studies have been conducted to determine the effect of heavy metal contamination on antibiotic resistance in wastewater discharged in slums. Therefore, the objectives of this study were; to determine the levels of heavy metals and antibiotic resistance patterns of *E. coli* in wastewater discharged in selected slums of Kawempe division, Kampala, and determine the effect of heavy metals on the antibiotic resistance patterns of *E. coli.* The study findings provide information on the current status of heavy metal contamination and antibiotic resistance patterns in wastewater discharged in slums of Kawempe division, Kampala. The findings also indicate the role of heavy metals on the prevalence of antibiotic resistance in wastewater.

## Materials and methods

### Study area

**Fig. 1:**
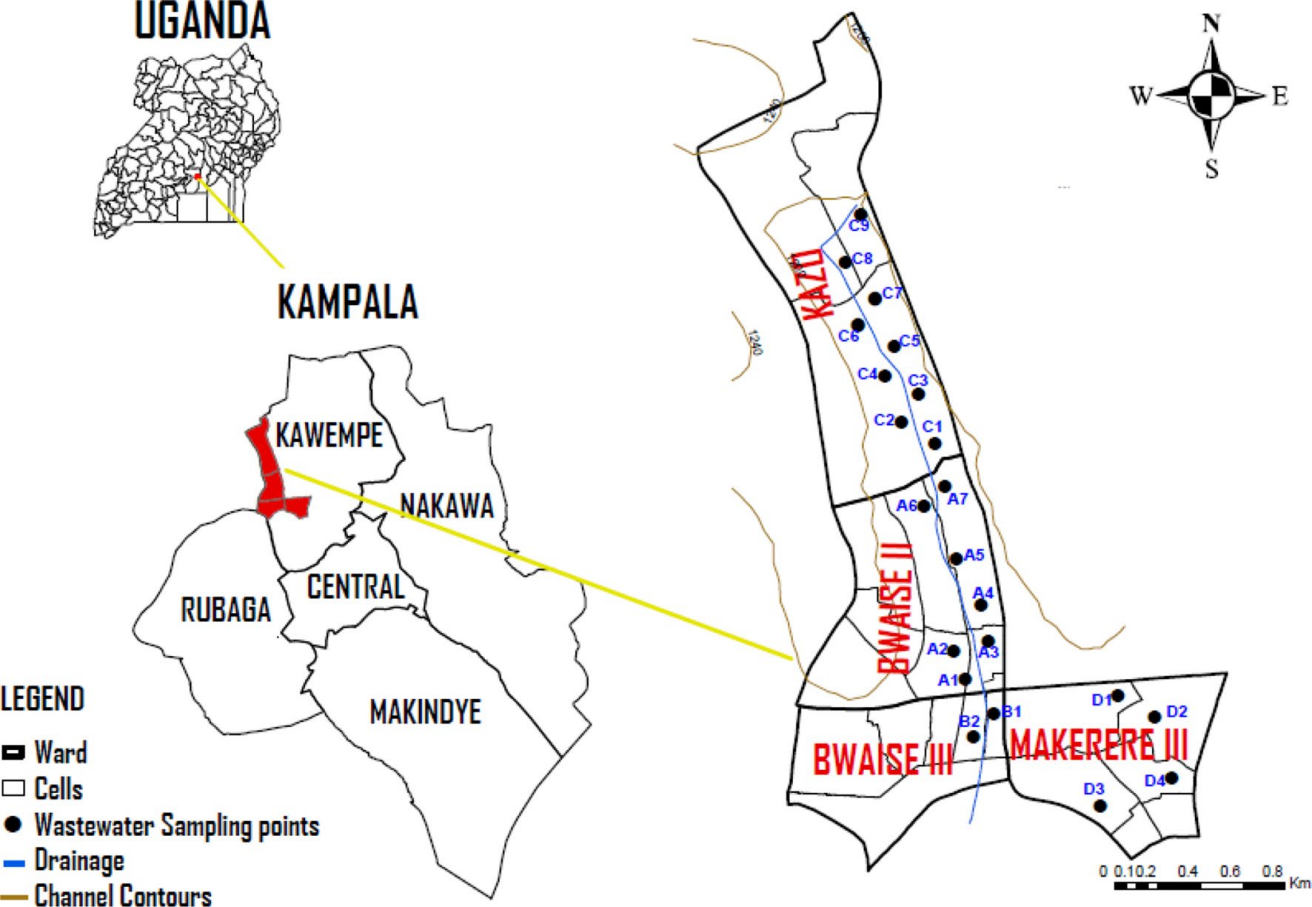
Wastewater sampling points in Kawempe division, Kampala

The study was conducted in Kawempe which constitutes one of the five administrative divisions of Kampala the capital city of Uganda. Kawempe division is a peri-urban setting located at 00^O^22’45” N 32^O^33’27” E in the northwestern corner of Kampala city, bordered by Wakiso district in the west, Nakawa division in the southeast, Central division in the south and Rubaga division in the southwest (Fig. 1). Kawempe is characterized by poor waste management and drainage systems, poor housing conditions and limited access to clean water, as well as numerous fuel stations and motor workshops [35, 36]. It has a population of 338,665 with 52% as women and about 94,202 households. Its climate is tropical rainforest featuring wet and dry seasons with average rainfall ~ 1200 mm/year [37, 38].

Kawempe has about 15 informal settlements (slums) accounting for about 85.8% (290,500 of 338,665) of its population, and majority of the unlined pit latrines (38%) of the total pit latrines a predominant sanitation technology in Kampala [35].

### Study design

The study design was both experimental and a case study. Kawempe division was selected because of the highest unimproved sanitation incidences where excreta are not hygienically separated from human contact compared to other divisions of Kampala. Slums of Bwaise II, Bwaise III, Kazo, and Makerere III were purposively sampled because of their highest levels of unimproved sanitation compared to other parishes in Kawempe division [35]. Wastewater sampling points were purposively sampled at the intersection points of wastewater emerging from at least two households, near the walkway, waste dumping sites, and feeding points of the free-range domestic animals (hens, ducks).

### Pilot survey

A pilot survey was conducted for four days in the slums of Bwaise II, Bwaise III, Kazo, and Makerere III to identify wastewater sampling points. The GPS coordinates and photos of the selected sites were captured using the Kobo Collect data collection tool (Additional file 1).

### Collection of wastewater samples

Wastewater samples were collected in duplicate from 22 identified sources in slums of Bwaise II (7), Bwaise III (2), Kazo (9), and Makerere III (4) potentially contaminated with heavy metals and *E. coli*. The samples were collected early morning (6:30 am-8:00 am) because of the more stabilized pH and temperature pronounced at that time of the day. Samples were collected in well-labeled sterile 50 ml and 500 ml screw-capped plastic bottles, transported to the laboratory in ice-cooled boxes at 4 ^O^C, and stored in a refrigerator at 4 ^O^C prior analysis. Before analysis, the duplicate wastewater samples from each sampling point were mixed to form a composite sample. The 50 ml samples were used to isolate *E. coli* in the Microbiology laboratory at the Department of Plant Sciences, Microbiology and Biotechnology, Makerere University while the 500 ml samples were used to determine the levels of heavy metals at the Government Analytical Laboratory in Wandegeya, Kampala-Uganda.

### Determination of the levels of heavy metals

The levels of heavy metals lead (Pb), mercury (Hg), cadmium (Cd), chromium (Cr), and arsenic (As) were analyzed by the inductively coupled plasma mass spectroscopy (ICP-MS, Agilent 7700, USA) following the HJ776-2015 method and the ISO/IEC 17025:2017 requirements. The composite wastewater samples were filtered using a 0.45 μm pore filter membrane. To 25 ml of the filtrates, 1 ml of 30% hydrogen peroxide was added followed by 1 ml of concentrated nitric acid. The mixture was then digested at 180 ^O^C for 15 min. The digests were diluted by addition of 50 ml of de-ionized water and the concentrations (ppb) determined using inductively coupled plasma mass spectroscopy [39]. This was replicated 10 times to determine the levels of heavy metals in the wastewater filtrates from each sampling point. A mixed standard solution including all the heavy metals (Pb, Hg, Cd, Cr, As) and reagent blanks was carried out through digestion and analyzed as part of the quality control protocol.

### Isolation of *E. coli* from wastewater

Ten-fold serial dilution was performed on wastewater samples using distilled water. For every sample, 100 µl of the 10^− 5^ serial dilution was surface plated on MacConkey agar (Jijan Babio Biotechnology Co. Ltd.) and incubated at 37 ^O^C for 48 h. A reference strain (*E. coli* ATCC 25922) was surface plated as a positive control, and MacConkey agar with no inoculum as a negative control. *E. coli* colonies were identified by their morphological similarity to the reference strain which grow as pink colonies surrounded by an area of precipitated bile salts on MacConkey agar [40]. The selected *E. coli* colonies were sub-cultured in Luria-Bertani broth (Scharlab, Spain) at 37 ^O^C for 24 h in an orbital shaker at 125 rpm to obtain pure cultures, and later stored at 4 ^O^C prior to screening for antibiotic resistance [33, 41].

### Determination of antibiotic resistance patterns

Agar well diffusion method was used to test for the susceptibility of *E. coli* to antibiotics [42]. The antibiotic stock solutions of tetracycline (TET, 30 µg/ml), amoxicillin (AMX, 30 µg/ml), ceftriaxone (CFT, 30 µg/ml), and ciprofloxacin (CIP, 5 µg/ml) were prepared. Pure cultures of *E. coli* were standardized by dilution using freshly prepared Luria-Bertani (LB) broth until a consistent OD_600_ = 0.5 equivalent to 3 × 1000^8^ CFU/mL was obtained. LB agar plates were prepared and 100 µl of the pure culture surface plated. This was followed by creating wells on the agar plates using a sterile micropipette tip, addition of 50 µl of the stock antibiotics, and incubation at 37 ^O^C for 24 h. The diameter (mm) of zone of inhibition was measured and interpreted as resistant, intermediate, or susceptible according to the Clinical Laboratory Standard Institute (CLSI) guidelines for Enterobacterales [43]. During this study, all isolates that showed intermediate zone of inhibition were considered resistant. The multiple antibiotic resistance index (MARI) was also determined for each isolate as described by [44] using the formula below.

$${\rm{MARI}}\>{\rm{ = }}\>{{{\rm{No}}{\rm{.}}\>{\rm{of}}\>{\rm{antibiotics}}\>{\rm{the}}\>{\rm{isolate}}\>{\rm{is}}\>{\rm{resistant\,to}}} \over {{\rm{Total}}\>{\rm{number}}\>{\rm{of}}\>{\rm{antibiotics}}\>{\rm{tested}}\>{\rm{against}}}}$$

### Determination of the effect of heavy metals on antibiotic resistance patterns

This included assessing the relationship between the presence of heavy metals and prevalence of antibiotic resistance in the collected wastewater samples, and determining the potential of heavy metals to induce antibiotic resistance in *E. coli* by the microdilution method [45]. In the latter case, the antibiotic susceptible *E. coli* isolates from the sampling points where no heavy metals were detected were used in this study. These were re-cultured in freshly prepared LB broth at 37 ^O^C for 24 h with shaking at 200 rpm, and later standardized by dilution using freshly prepared broth until a consistent OD_600_ = 0.5 equivalent to 3 × 1000^8^ CFU/ml was obtained. Stock solutions of heavy metals (Pb, Cd, Hg) were prepared from salts of Pb(NO3)_2_ (Scharlab, Spain), CdCl_2_ (BDH Chemicals Ltd Poole, England), and Hg(NO3)_2_ (BDH Chemicals Ltd Poole, England), respectively. The molecular weight of the salt and the atomic weight of the heavy metal was used to calculate the weight composition (P) of the heavy metal in the respective salt. This was used to determine the amount of the salt (Y) needed to prepare a stock solution with the desired amount of the heavy metal (M) using the formula below [46].

$$\:\left(P\right)\:X\:\left(Y\right)\:=\:\left(M\right)$$

$$\>{\rm{Y}}\>{\rm{ = }}\>{{\left( {\rm{M}} \right)} \over {\left( {\rm{P}} \right)}}$$

The µg equivalent of the salt weighed was dissolved in 1 ml of distilled water to make a one-time (1X) stock solution of the heavy metal salt. The stock solutions were prepared from the average amount of the heavy metals detected in wastewater samples. The antibiotic susceptible isolates were cultured in 200 µl of LB broth containing 10 µl of stock antibiotics (tetracycline, amoxicillin, ceftriaxone at 30 µg/ml; and ciprofloxacin at 5 µg/ml), or a combination of stock antibiotics (10 µl) and stock heavy metals (10 µl). The plates were then incubated at 37 ^O^C for 48 h. The growth of each isolate exposed to either antibiotics or a combination of antibiotics and heavy metals was assessed by measuring their OD_600_ using the iMark microplate absorbance reader (Bio-Rad, USA). A negative control excluded exposure of isolates to either the antibiotics or heavy metals. The resultant microbial growth patterns were used to determine the effect of heavy metals on antibiotic resistance in *E. coli*.

### Data analysis

Raw data sorting and descriptive statistics were performed using MS Excel 2019. The antibiotic resistance patterns of *E. coli* isolates were compared using chi-square tests. Linear regression was used to assess the relationship between heavy metals and antibiotic resistance patterns. T-test was used to compare the resistance patterns of *E. coli* on exposure to antibiotics against a combination of antibiotics and heavy metals. Comparisons were considered significantly different at a level of 95% CI (*p* ≤ 0.05). The linear regression, T-test, chi-square test, and the graphical presentation of the data were performed using GraphPad Prism 8.0.1.

## Results

### Levels of heavy metals in wastewater

The pollution levels of heavy metals in wastewater from slums of Bwaise II, Bwaise III, Kazo and Makerere III are presented in Table 1. Results showed that at least one heavy metal was detected in 50% (11 of 22) of the wastewater samples collected. Detectable levels of heavy metals were reported at sampling points A1, A2, A4 and A6 in Bwaise II; C1, C2, C3, C4 and C9 in Kazo; and D2 and D4 in Makerere III. The highest levels of Pb were detected in Bwaise II at sampling point A4, while Cd and As were highest in Kazo at sampling points C1 and C2, respectively. Levels of Hg and Cr were highest in Makerere III at sampling points D2 and D4, respectively.

**Table 1:** The mean ± sd levels of heavy metals (ppb) detected in wastewater samples from slums of Bwaise II, Bwaise III, Kazo and Makerere III

Sampling area	Sampling point	Heavy metals (LOD)				
		Hg (0.54)	Pb (0.35)	Cd (0.15)	Cr (0.5)	As (0.5)
Bwaise II	A1	4.21 ± 0.56	-	-	-	12.70 ± 0.04
	A2	-	-	-	34.14 ± 0.21	-
	A3	-	-	-	-	-
	A4	2.03 ± 0.09	783.84 ± 0.22	45.05 ± 0.08	-	-
	A5	-	-	-	-	-
	A6	-	-	-	5.11 ± 0.92	8.05 ± 0.14
	A7	-	-	-	-	-
Bwaise III	B1	-	-	-	-	-
	B2	-	-	-	-	-
Kazo	C1	-	-	61.03 ± 0.21	-	-
	C2	-	-	-	2.16 ± 0.11	647.58 ± 0.94
	C3	-	13.22 ± 0.17	-	4.39 ± 0.22	-
	C4	-	-	-	15.92 ± 0.03	-
	C5	-	-	-	-	-
	C6	-	-	-	-	-
	C7	-	-	-	-	-
	C8	-	-	-	-	-
	C9	1.06 ± 0.02	-	3.06 ± 0.11	2.04 ± 0.01	-
Makerere III	D1	-	-	-	-	-
	D2	12.39 ± 0.07	-	-	-	-
	D3	-	-	-	-	-
	D4	-	274.12 ± 0.06	26.36 ± 0.33	73.93 ± 0.17	16.02 ± 0.03

LOD; Level of detection, SD; Standard Deviation, (-); no detectable heavy metals

### Antibiotic resistance patterns of *E. coli* in wastewater samples

Results for *E. coli* isolates in wastewater are presented in Table 2. Wastewater samples from all sampling points showed growth of *E. coli* on MacConkey agar plates. A total of 232 out of 769 isolates were identified as *E. coli* based on their morphological similarity with a reference strain.

**Table 2:** Number of isolates identified as *E. coli* from wastewater samples

Sampling area (*n*)	Total isolates	E. coli isolates
Bwaise II (7)	274	93
Bwaise III (2)	67	16
Kazo (9)	324	82
Makerere III (4)	104	41
**Total**	**769**	**232**

n = number of wastewater samples

Results for the susceptibility tests of *E. coli* isolates from the different slum areas under this study are presented as Additional file 2. Overall, 72.8% (169 out of 232) of the *E. coli* isolates from the different slum areas were resistant to at least one of the four antibiotics (Table 3).

**Table 3:** Percentage antibiotic resistance and the multiple antibiotics resistance index (MARI) of *E. coli* isolated from wastewater from the slums of Kawempe

Sampling Area	Resistant E. coli isolates (*n*)	% Resistance	MARI
Bwaise II	67(93)	72.0	0.28
Bwaise III	8(16)	50.0	0.23
Kazo	58(82)	70.7	0.32
Makerere III	36(41)	87.8	0.45

n = number of isolates screened for resistance

**Fig. 2:**
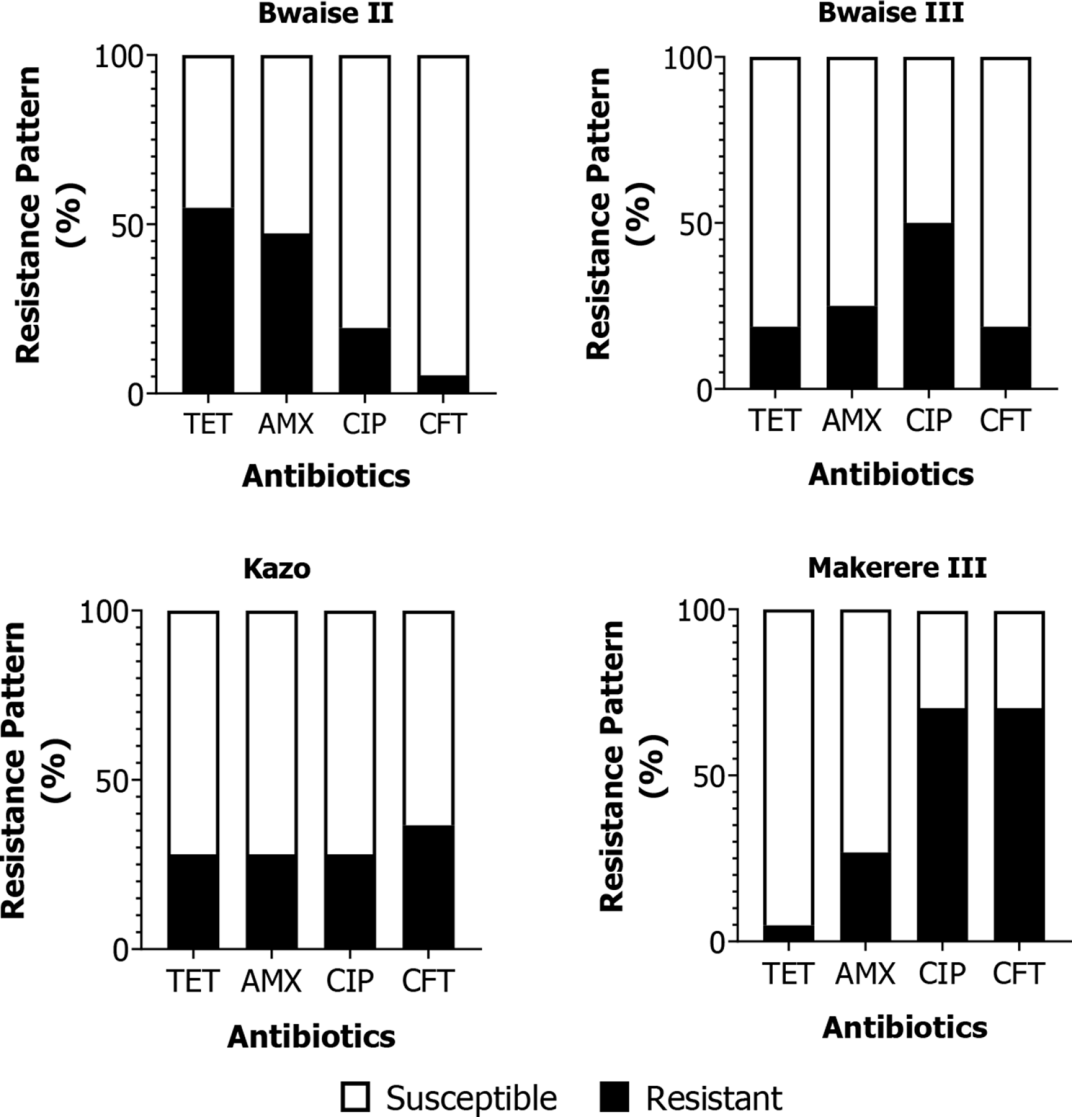
Antibiotic resistance patterns of *E. coli i*solated from wastewater samples

The resistance pattern for each antibiotic varied significantly (*p* < 0.000) for all *E. coli* isolates from the different slum areas. The proportions of *E. coli* resistant to tetracycline, ceftriaxone, amoxicillin, and ciprofloxacin were 34.1%, 28.9%, 35.3%, and 34.5%, respectively. The trends of resistance patterns for *E. coli* isolates from Bwaise II, Bwaise III, Kazo, and Makerere III were TET > AMX > CIP > CFT; CIP > AMX > TET = CFT; CFT > TET = AMX = CIP; and CFT = CIP > AMX > TET, respectively (Fig. 2).

### Effect of heavy metals on antibiotic resistance patterns

**Fig. 3:**
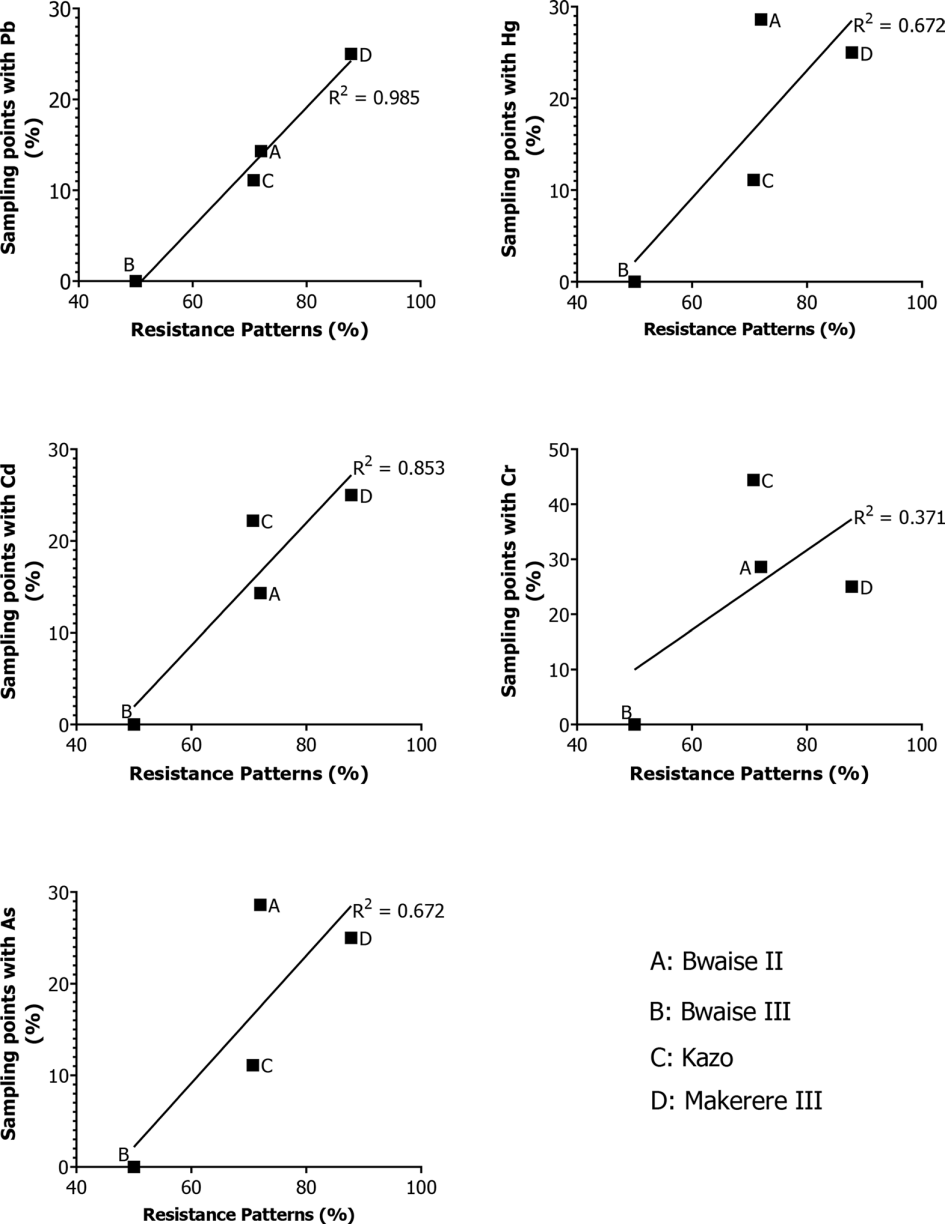
Relationship between the presence of heavy metals and antibiotic resistance patterns of *E. coli* in wastewater

**Fig. 4:**
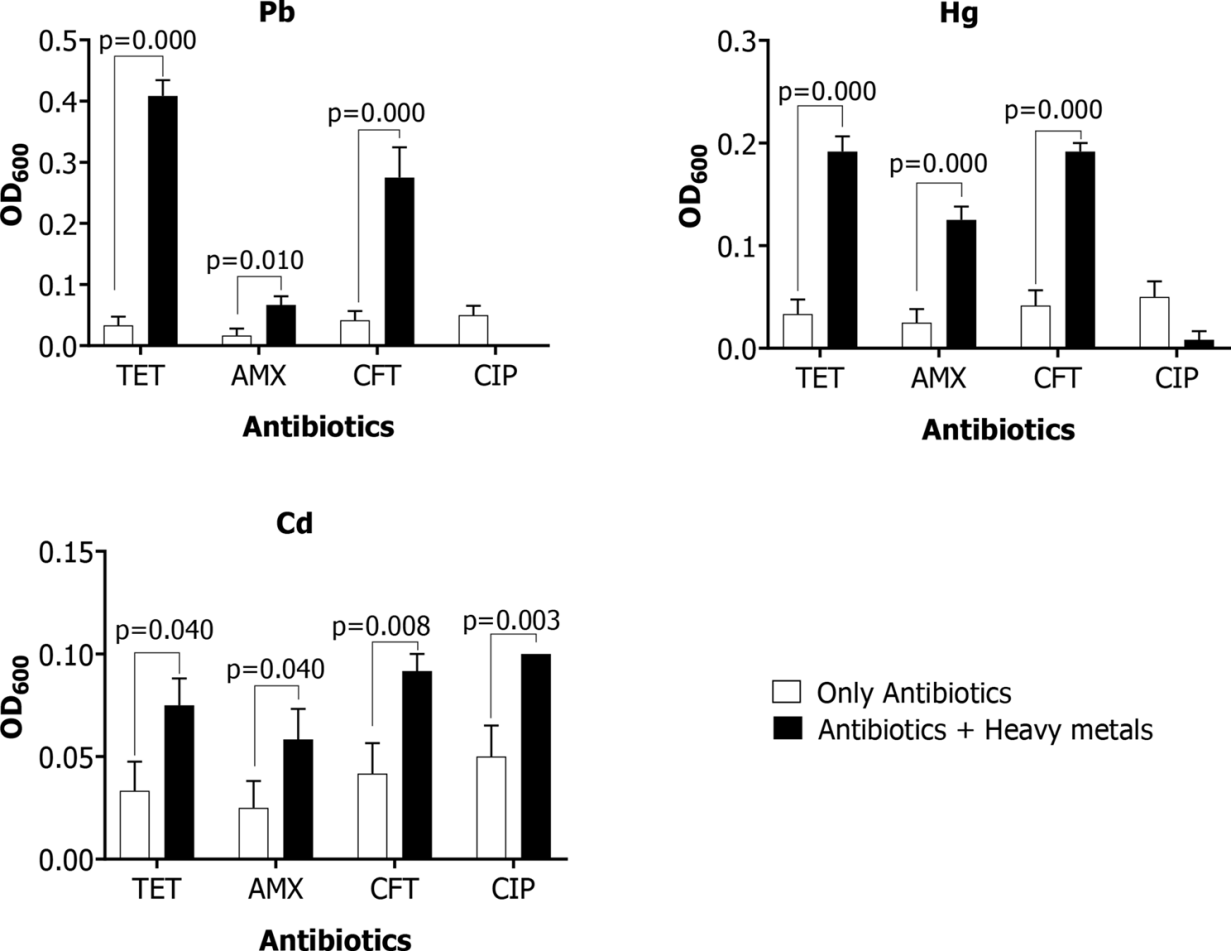
Growth of *E. coli* in LB broth supplemented with antibiotics only and a combination of antibiotics with heavy metals at OD_600nm_

Results showed positive correlation between the presence of heavy metals namely; Pb (R^2^ = 0.985), Hg (R^2^ = 0.672), Cd (R^2^ = 0.853), Cr (R^2^ = 0.371), As (R^2^ = 0.672) and the antibiotic resistance patterns of *E. coli* from sampling points at the different slum areas investigated (Fig. 3). Results for OD_600_ showing growth of *E. coli* in media supplemented with a combination of antibiotics (tetracycline, amoxicillin, ceftriaxone, ciprofloxacin) and heavy metals; Pb (77.41 µg/ml), Hg (1.44 µg/ml), and Cd (10.21 µg/ml), and growth supplemented with antibiotics only are presented as Additional file 3. There was a significant increase (*p* < 0.05) in the growth of *E. coli* isolates (*n* = 12) cultured in media supplemented with a combination of antibiotics and heavy metals compared to growth supplemented with antibiotics only (Fig. 4).

## Discussion

Slum dwellers face significant infrastructure and public health challenges like poor housing and drainage, inadequate sanitation, and limited access to clean water [6, 13]. This study revealed that heavy metals were present and the magnitude of their levels in wastewater collected from the different slum areas of Kawempe division varied. The variations could have been due to differences in soil components and wastewater generated at the different sampling sites.

Lead was recorded highest in Bwaise II at sampling point A4, located downstream of the dumping site of wastes generated from the market in Jambula cell, which suggest the possible source of the high Pb levels detected in wastewater. Moreover [47, 48], in their studies reported that the source of Pb pollution in the environment is usually from electronic and battery wastes, corrosion of old buildings with lead paints, and runoffs from bituminous paving on roads. The highest levels of Cd and As were reported in wastewater samples from Kazo at sampling points C1 and C2, respectively. At the upstream end of sampling point C1 in Kazo-Angola cell, there is an Agri-processing factory, and household solid waste dumping site which could be the source of high Cd levels detected in wastewater at the sampling point. At the upstream end of sampling point C2 in Kazo-Angola cell, brick making activities, and household wastes burning spots were observed near the drainage systems. In addition to runoffs from a water spring and garden near the sampling point, these could have served as the sources of arsenic. The observed potential sources of As and Cd are in line with what [49, 50] reported in their study as the source of arsenic and cadmium pollution, i.e., combustion of fossil fuel, incineration of municipal and industrial wastes, human activities such as mining, land application of sewage sludge, and fertilizer EPA. Levels of Hg and Cr were highest in wastewater from Makerere III at sampling points D2 in Kibbe cell and D4 in Sebina cell, respectively. The runoffs through metallic case and carpentry workshops, and waste dumping sites in the neighboring areas of the sampling points could be the potential sources of the high Cr and Hg levels detected. Moreover [51], in their study reported metal coating, dyes production for painting, wood protection materials, textile industry, chemical production, fluorescent light lamps, and fungicides as sources of Hg and Cr pollution in the environment.

The levels of Pb detected in Bwaise II and Makerere III at sampling points A4 and D4, respectively were higher than permissible limits by WHO (10 ppb) and NEMA (100 ppb), whereas at sampling point C3 in Kazo they were higher than the limits by WHO only. The levels of Cd in Bwaise II, Kazo, and Makerere III at sampling points A4, C1 and C9, and D4, respectively exceeded the permissible limits by the WHO (3 ppb). Only Cr levels detected in Makerere III at sampling point D4 exceeded the permissible limits by WHO (50 ppb) and NEMA (50 ppb), whereas Hg exceeded the permissible levels by WHO (1 ppb) in wastewater samples from Bwaise II, Kazo, and Makerere III at sampling points A1 and A4, C9, and D2, respectively. Heaps of wastes were observed at all wastewater sampling points or at the upstream ends where the levels exceeded the permissible limits. These suggest spill offs from the wastes as the potential source of heavy metal pollution in slums of Kawempe division.

In this study, 72.8% of *E. coli* isolates were resistant to at least one of the four antibiotics tested. This was in agreement with findings by [52] who reported resistance patterns of *E. coli* to at least one of the 16 antimicrobial agents screened in Africa between 2005 and 2018 that ranged from 33.3 to 100%. Worth noting is that 43% of people living in slums do self-medication using antibiotics [53]. In addition, 30–90% of the ingested antibiotics are excreted unchanged [54] which end up in wastewater. Moreover, antibiotics and various personal care products used, as well as anthropogenic activities contribute to a build-up of antibiotic selection pressure in wastewaters [55].

A study by [56] described the average multiple antibiotic resistance index (MARI) value as an indicator of the level of contaminants in the environment where the bacteria are isolated. This study revealed that the average MARI value of isolates varied amongst slums. Moreover, wastewater samples from Bwaise III with heavy metals below the level of detection had isolates with the lowest MARI value compared to the other slums with detectable levels. This finding relates with findings by [57] and [58] who reported differences in the MARI values for isolates from hospital wastewater and non-hospital wastewater. In addition, there was a positive correlation between the presence of heavy metals in wastewater and antibiotic resistance patterns (R^2^ = 0.371–0.985) across the slums, signifying the role of heavy metals in the build-up of antibiotic selection pressure.

Some heavy metals at low concentrations are reported to be essential elements in different biochemical processes and may become toxic at high concentrations [59]. In addition, prolonged exposure to heavy metals has been reported to have effects on the composition and activity of microbial communities by selecting for metal resistance as well as co-selection for resistance to antibiotics through activation of efflux pumps, enzyme detoxification, and alteration of cellular targets [60].

During this study, supplementing the growth media with a combination of heavy metals and antibiotics significantly (*p* < 0.05) counteracted the antibiotic effects on *E. coli* leading to an increase in their growth. This relates well with findings by [61] who reported a significant increase (*p* < 0.05) in the minimum inhibitory concentrations of ampicillin against pathogenic bacteria collected from sites close to metal mining activities compared to the control sites, and [62] who reported a significant increase in bacterial growth in heavy metal plus antibiotics supplemented media. The exposure to Pb, Cd, and Hg could have activated cross resistance mechanism leading to mutations in the ribosomes which in turn change the configuration of the binding sites for tetracycline [63]. Also, this exposure could have activated the co-resistance mechanism inducing the expression of beta-lactam genes that code for beta-lactamase enzyme which hydrolyze the beta-lactam ring in ceftriaxone and amoxicillin [64]. Besides, resistance to ciprofloxacin was only increased with exposure to Cd which could have resulted from the higher interaction capacity with the gyrase enzyme unlike Pb and Hg or the high concentration of Cd that interfered with Mg^2+^ co-factor functions [65, 66]. Co-resistance occurs when two or more resistance genes are present on the same mobile genetic element, such as plasmids, transposons hence inducing multiple resistance mechanisms. Moreover, heavy metal resistance genes and antibiotic resistance genes have been reported to co-occur on the same mobile genetic elements [31]. Cross-resistance on the other hand occurs when a single mechanism such as mutation in the target sites simultaneously induces resistance to different stress agents. This is supported by findings of [67] who reported that more than 75% of the bacteria isolates with high tolerance to heavy metals were resistant to some antibiotics yet very few tested positive for antibiotic resistance genes; and findings of [68] who reported an association between tetracycline and heavy metal resistance.

Our study findings, therefore, elaborated the role of heavy metal pollution with regard to the prevalence of antibiotic resistance in *E. coli*. With more than 80% of the world’s wastewater generated being discharged into the environment without treatment, the public health is at risk of increased antibiotic resistant infections [36, 69]. Surface water sources such as rivers, lakes, and wells being major sinks for the wastewater discharged into the environment [16], water quality will increasingly be compromised by antibiotic resistant bacteria. In addition, wastewater is also used in agriculture for irrigation farming [70] which predisposes plants to contamination with antibiotic resistant pathogenic bacteria.

## Conclusions

The present study highlights the presence of heavy metals in wastewater collected from slums of Kawempe Division, Kampala with Pb, Hg, and Cd at levels higher than permissible limits set by WHO. The study further reported high resistance patterns (72.8%) of *E. coli* isolated from wastewater samples to tetracycline, amoxicillin, ceftriaxone, and ciprofloxacin. The findings also reported the significant role of Pb, Cd, and Hg in inducing resistance of *E. coli* to different classes of antibiotics, which represent a major public health concern. This therefore calls for proper wastewater management to contain the prevalence of antibiotic resistance in pathogenic microorganisms.

This study recommends more research involving larger sample size and seasonal variations as well as other divisions of Kampala to obtain more data regarding heavy metal pollution, and antibiotic resistance. Future studies may also focus on the long-term exposure of pathogenic bacteria to heavy metals and how it affects the biochemical pathways to ascertain their AMR mechanisms.

## Data Availability

All relevant data are reported in the manuscript.

## Abbreviations

AMR: Antimicrobial resistance
AMX: Amoxicillin
CFT: Ceftriaxone
CFU: Colony-Forming Unit
CIP: Ciprofloxacin
DGAL: Directorate of the Government Analytical Laboratory
DNA: Deoxyribonucleic acid
GPS: Global Positioning System
ICP-MS: Inductively Coupled Mass spectroscopy
KCCA: Kampala Capital City Authority
MARI: Multiple Antibiotic Resistance Index
NEMA: National Environment Management Authority
RPM: Revolutions Per Minute
TET: Tetracycline
UBOS: Uganda Bureau of Statistics
WHO: World Health Organization
